# Targeting the RhoGEF βPIX/COOL-1 in Glioblastoma: Proof of Concept Studies

**DOI:** 10.3390/cancers12123531

**Published:** 2020-11-26

**Authors:** Kate Connor, David W. Murray, Monika A. Jarzabek, Nhan L. Tran, Kieron White, Patrick Dicker, Kieron J. Sweeney, Philip J. O’Halloran, Brian MacCarthy, Liam P. Shiels, Francesca Lodi, Diether Lambrechts, Jann N. Sarkaria, Raymond M. Schiffelers, Marc Symons, Annette T. Byrne

**Affiliations:** 1 Department of Physiology and Medical Physics, Royal College of Surgeons in Ireland, Dublin 2, Ireland; kateconnor@rcsi.ie (K.C.); david13murray@gmail.com (D.W.M.); monika.jarzabek@gmail.com (M.A.J.); kieronwhite@rcsi.ie (K.W.); KieronJSweeney@rcsi.ie (K.J.S.); philohalloran@rcsi.ie (P.J.O.); brianmaccarthy@gmail.com (B.M.); liamshiels@rcsi.ie (L.P.S.); 2 Department of Cancer Biology and Neurological Surgery, Mayo Clinic Arizona, Scottsdale, AZ 85054, USA; Tran.Nhan@mayo.edu; 3 Epidemiology & Public Health, Royal College of Surgeons in Ireland, Dublin 2, Ireland; patdicker@rcsi.ie; 4 National Neurosurgical Department, Beaumont Hospital, Dublin 9, Ireland; 5 Center for Cancer Biology, Laboratory for Translational Genetics, Vlaams Instituut voor Biotechnologie (VIB), B-3000 Leuven, Belgium; francesca.lodi@kuleuven.be (F.L.); diether.lambrechts@kuleuven.be (D.L.); 6 Department of Radiation Oncology, Mayo Clinic, Rochester, MN 55905, USA; Sarkaria.Jann@mayo.edu; 7 Department of Clinical Chemistry and Haematology, University Medical Center Utrecht, 100 3584 Utrecht, The Netherlands; R.Schiffelers@umcutrecht.nl; 8 Department of Oncology & Cell Biology, Feinstein Institute for Medical Research at North Shore-LIJ, Manhasset, NY 11030, USA; MSymons@northwell.edu

**Keywords:** glioblastoma, bevacizumab resistance, anti-invasive therapy, RhoGEF, Beta-Pix/COOL-1, ARHGEF7

## Abstract

**Simple Summary:**

Glioblastoma (GBM) is an incurable disease with a 14-month average life-expectancy following diagnosis, and clinical management has not improved in four decades. GBM mortality is due to rapid tumour growth and invasion into surrounding normal brain. Invasive cells make complete surgical removal of the tumour impossible, and result in disease relapse. Thus, it is imperative that any new treatment strategy takes these invading cells into consideration. Bevacizumab (Bev), which prevents the formation of new blood vessels, is an FDA approved therapy, but it has failed to increase overall survival in GBM and has even been shown to increase tumour invasion in some cases. Complementary anti-invasive therapies are therefore urgently required to enhance bevacizumab efficacy. We have identified βPIX/COOL-1, a RhoGEF protein which plays an important role in GBM cell invasion and angiogenesis and could be a useful target in this setting.

**Abstract:**

Glioblastoma (GBM), a highly invasive and vascular malignancy is shown to rapidly develop resistance and evolve to a more invasive phenotype following bevacizumab (Bev) therapy. Rho Guanine Nucleotide Exchange Factor proteins (RhoGEFs) are mediators of key components in Bev resistance pathways, GBM and Bev-induced invasion. To identify GEFs with enhanced mRNA expression in the leading edge of GBM tumours, a cohort of GEFs was assessed using a clinical dataset. The GEF βPix/COOL-1 was identified, and the functional effect of gene depletion assessed using 3D-boyden chamber, proliferation, and colony formation assays in GBM cells. Anti-angiogenic effects were assessed in endothelial cells using tube formation and wound healing assays. In vivo effects of βPix/COOL-1-siRNA delivered via RGD-Nanoparticle in combination with Bev was studied in an invasive model of GBM. We found that siRNA-mediated knockdown of βPix/COOL-1 in vitro decreased cell invasion, proliferation and increased apoptosis in GBM cell lines. Moreover βPix/COOL-1 mediated endothelial cell migration in vitro. Mice treated with βPix/COOL-1 siRNA-loaded RGD-Nanoparticle and Bev demonstrated a trend towards improved median survival compared with Bev monotherapy. Our hypothesis generating study suggests that the RhoGEF βPix/COOL-1 may represent a target of vulnerability in GBM, in particular to improve Bev efficacy.

## 1. Introduction

Glioblastoma (GBM), a heterogeneous and lethal brain tumour, remains an incurable malignancy, capable of rapidly developing resistance to treatment [1]. Despite significant progress in molecular subtyping and an improved understanding of the plasticity of GBM heterogeneity [2], clinical management has not significantly improved in several decades [3]. The Stupp protocol, consisting of maximal safe surgical resection, radiotherapy and chemotherapy with temozolomide (TMZ) persists as the standard of care [4] for this treatment refractory disease. Notably, tumour-associated angiogenesis is a distinct hallmark of the disease, with the VEGF-signaling axis a now well described target of intervention [5,6]. Bevacizumab (Bev), an anti-VEGF antibody, has been shown to improve time to progression for newly diagnosed GBM [7]. Despite the improved time to progression for patients, and beneficial anti-oedematous effects [8], Bev-treated GBM tumours rapidly adapt and develop resistance, even sometimes evolving to a more aggressive phenotype [9,10]. Increased δ-catenin [11], stem cell marker [10] and matrix metalloproteinase family member expression [12], augmented tyrosine kinase receptor (TKR) activity [13] and indeed a shift towards a more aggressive mesenchymal phenotype [10] are among the consequences of Bev therapy in GBM. Bev has further been shown to enhance GBM cell invasion [14,15] with distant infiltrative disease sometimes observed following treatment [16,17]. Overall, Bev has failed to elicit a benefit in overall survival (OS) when added to standard of care. This has now been demonstrated in two Phase III randomized clinical trials RTOG-0825 (NCT00884741) and AVAGlio (NCT00943826) [18,19].

RhoGEFs activate and control Rho GTPase protein activity by facilitating GDP exchange [20]. These Rho GTPases function as molecular switches, and are key mediators of angiogenesis signaling, cytoplasmic plasticity, cell polarity, vesicle trafficking, cytoskeletal regulation and gene transcription [21,22]. Rho GEFs activate proteins such as Cdc42, GIT, PAK and Rac which underpin key tumour cell invasion processes such as formation of lamelopodia, invadiopodia and focal adhesions [23]. Several studies have also provided evidence to support a role for the GEF proteins Ect2, DOCK7 and PDZ-RhoGEF in GBM cell invasion [21,24,25]. Moreover, a role for the GEF LARG in GBM migration and invasion has recently been elucidated [26]. Latterly, GEF-H1 has also been implicated in the mechanism of tumour-treating fields (TTF), a novel treatment modality that has recently been approved in the GBM setting [27]. TTF employs non-invasive electrical fields (200 kHz) to interrupt spindle fiber formation and inhibit tumour cell mitosis via tubulin and septin complex disruption. TTF further induces microtubule alterations resulting in GEF-H1 activation and subsequent disruption of cell migration [28]. Collectively, these data indicate that GEF proteins underpin the invasive capacity of GBM cells and may further play a role in mediating treatment response.

As mentioned, RhoGEF signaling also plays a key role in angiogenesis and vascular biology processes. For example, VEGF activation of Rho GTPase signaling is now well documented [22]. Moreover, Rho GTPase and RhoGEF proteins are known to promote angiogenesis by mediating endothelial cell migration [29]. In addition, the activity of several RhoGEFs underpin many of the cellular alterations induced by Bev treatment. For example, the activity of GEFs such as Vav2 [30] and P-Rex1 [31] are activated following anti-angiogenic therapy. Moreover, the GEFs Tuba and GEF-H1 play essential roles in vascular permeability and lumen formation [32]. We have also previously shown that the RhoGEF DOCK7 mediates cMET-induced GBM invasion, the latter being a well-established pathway of VEGFR-inhibitor resistance [13,24].

Overall, as RhoGEFs are mediators of key components in Bev resistance pathways, and are involved in tumour cell and Bev-induced invasion [14,15], we hypothesised that targeting Rho GTPase signaling might be an effective anti-invasive/anti-angiogenic therapeutic strategy. To this end, we first sought to identify GEFs with enhanced expression in the leading edge of GBM patient tumours. As the RhoGEF βPix/COOL-1 was found to upregulated in the invasive tumour edge, we subsequently studied its functional role in GBM and human endothelial cells using in vitro models. Finally, we performed a proof-of-concept in vivo study to study the effect of a novel treatment regimen comprised of Bev and a nanoparticle gene silencing system (RGD-NP) loaded with βPix/COOL-1-targeting siRNA. Our hypothesis generating study suggests that targeted inhibition of GEF signaling might improve VEGF inhibitor response.

## 2. Results

### 2.1. βPix/COOL-1 Plays a Key Role in GBM Cell Invasion

**Figure 1:**
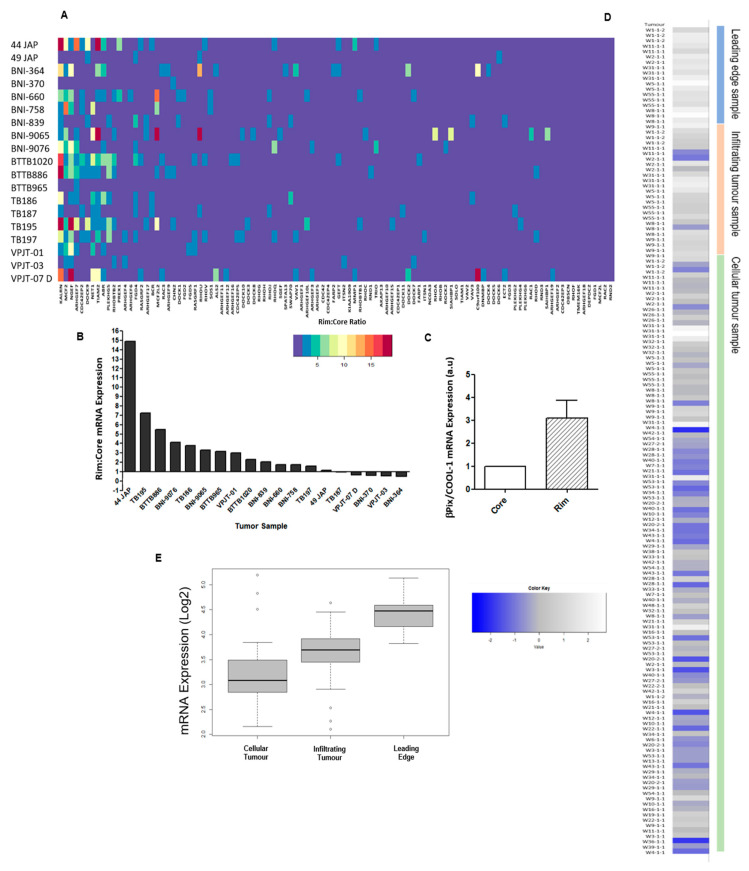
Rim:Core ratio analysis of tumours identifies βPIX/COOL-1 as being overexpressed in invasive GBM cells compared to tumour core. (**A**) Rim to Core mRNA expression ratio of all GEF family proteins in 19 tumour specimens; (**B**) From 19 independent GBM specimens, stationary (core) and invasive (rim) cells were assessed for relative βPIX/COOL-1/ARHGEF7 mRNA signal intensity, which was expressed as a ratio of rim to core (from NCBI Gene Expression Omnibus GSE12689); (**C**) Average Rim: Core βPIX mRNA expression across all tumour samples; (**D**) ARHGEF7/βPIX/COOL-1 mRNA expression in leading edge, infiltrating tumour and cellular tumour core samples (defined by reference histology) across 42 tumour samples from the IvyGap database (GSE107559) (**E**) Boxplot illustrating βPIX/COOL-1/ARHGEF7 mRNA expression in leading edge, infiltrating tumour and cellular tumour core samples from GSE107559. Wilcoxon signed-rank test, *p* < 0.05 deemed significant.

To identify RhoGEFs of functional importance in GBM invasion we first examined a panel of 19 treatment naïve GBM samples (NCBI, Gene Expression Omnibus GSE12689) for variations in the ratio of their leading edge (rim) to core mRNA expression of any of the RhoGEF family proteins [33]. We identified 12 GEFs which demonstrated a higher mRNA expression in the rim (invasive cells) of neoplastic tissue when compared to the tumour core (stationary cells) in more than 50% of tumours assessed. KALRN, MCF2/Dbl, NGEF/EPHEXIN, ARHGEF7/βPix/COOL-1, CDC42EP2, DOCK9, NET1, TIAM2, ABR, PLEKHG5, RhoBTB2 and PREX1 (Figure 1A and Figure S1) were identified as being increased at the tumour rim. We further showed that ARHGEF7/βPix/COOL-1 expression is increased at the invasive edge in 69% of tumours assessed, when compared with core mRNA expression level (Figure 1B). 10% of samples analysed displayed no difference in βPix/COOL-1 mRNA expression between the rim and core, and 21% of tumours displayed a lower core to rim mRNA ratio (Figure 1B,C).

ARHGEF7/βPix/COOL-1 mRNA expression was next assessed within the Ivy Glioblastoma Atlas Project RNAseq dataset (IvyGap GSE107559 [34]; *N* = 41 patients) (Figure 1D,E). Here, βPix/COOL-1 mRNA expression is significantly upregulated in samples derived from the leading tumour edge (15.57% of samples) compared to samples representing cellular tumour (24.59% of samples) (*p* = 1.22 × 10^−7^) and infiltrating tumour (19.67% of samples) (*p* = 1.446 × 10^−6^) (Figure 1E). Moreover, infiltrating tumour samples display significantly upregulated βPix/COOL-1 mRNA expression compared to cellular tumour samples (*p* = 0.004845) (Figure 1E). βPix/COOL-1 mRNA expression was further assessed in the single cell RNA sequencing (scRNA seq) dataset GSE84465 [35] (Figure S2). These data therefore provided a rationale to further study βPix/COOL-1 as a therapeutic target.

### 2.2. βPix/COOL-1 Mediates GBM Cell Invasion

**Figure 2:**
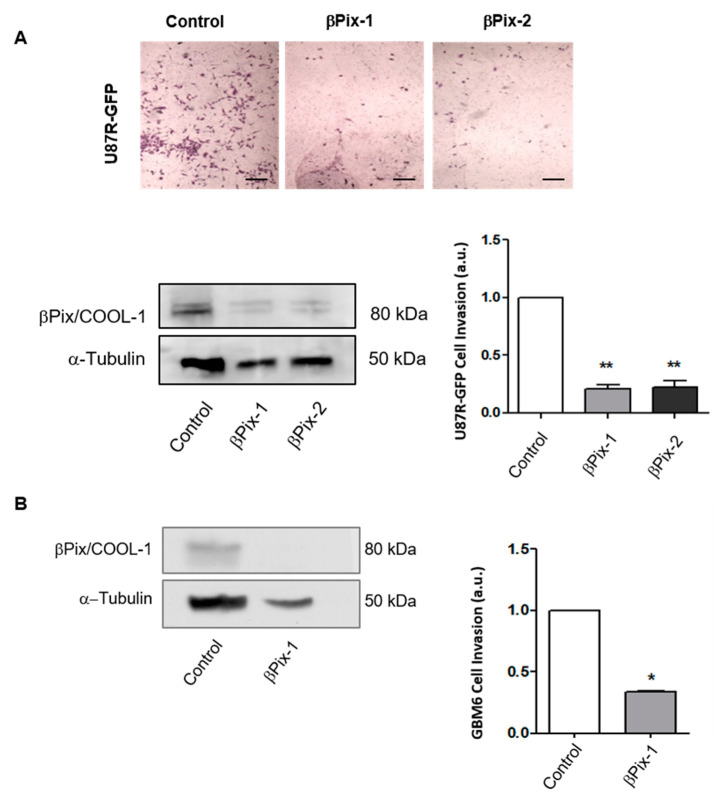
βPix/COOL-1 depletion inhibits cell invasion in two GBM cell lines. (**A**) The effect of beta-Pix knockdown using βPix-1 and βPix-2 siRNA duplexes (5 nM concentration) on GBM cell invasion in the U87R-GFP GBM cell line. Western blot analysis showing βPix/COOL-1 protein expression following siRNA knockdown in U87R-GFP cells confirms knockdown. (**B**) The effect of beta-Pix knockdown (βPix-1 siRNA) on PDX GBM6 cell invasion. Western blot analysis showing βPix/COOL-1 protein expression following siRNA knockdown in GBM6 cells. Tubulin was used as a loading control. Scrambled non-coding negative control (Cat#DSNC1, IDT Technologies, Coralville, IA, USA) was used as control (control) in both cell lines. Normalisation was performed by assigning the value Figure 1. and expressing all other values relative to it. Two-way ANOVA, * *p* < 0.05, ** *p* < 0.001 two-tailed *t*-test. The data shown are arbitrary units (a.u.) means ± s.d. (*N* = 3). Scale bar = 50 µm.

To assess the impact of increased βPix/COOL-1 rim mRNA expression and elucidate the role βPix/COOL-1 plays in this region of the tumour, we examined the effect of βPix/COOL-1 knockdown on the invasive capacity of two human GBM cell lines, U87R [36] and GBM6 [37]. Using a 3D Boyden chamber assay and serum as an attractant, we have shown that depletion of βPix/COOL-1 in U87R cells significantly decreased the number of invasive cells, with greater than a two-fold reduction in invasion (βPix-1: *p* = 0.0025; βPix-2: *p* = 0.0051). Two independent siRNA oligonucleotides were used to minimize the risk of RNA off-target effects (Figure 2A and Figure S2A) in the U87R cell line. The siRNA which induced greatest knockdown was further assessed in the GBM6 cell line and significantly inhibited invasive capacity of this cell line (*p* = 0.009) (Figure 2B and Figure S2B). This siRNA (βPix/COOL-1) was implemented for all subsequent GBM6 assays.

### 2.3. βPix/COOL-1 Knockdown Decreases GBM Proliferation and Viability and Enhances Apoptosis

**Figure 3:**
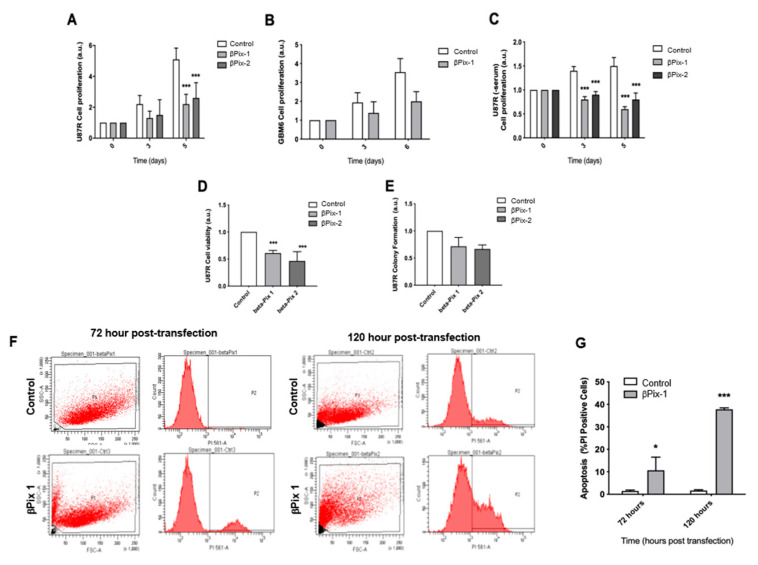
βPix/COOL-1 depletion inhibits proliferation of U87R-GFP and GBM6 GBM cells. (**A**,**B**) The effect of βPix/COOL-1 knockdown using two siRNA duplexes (βPix-1 and βPix-2) on GBM cell proliferation using U87R-GFP and patient derived GBM6 cells (**C**) The effect of beta-Pix knockdown (βPix-1 and βPix-2) on GBM cell proliferation in serum-free conditions using U87R-GFP cells. (**D**) The effect of βPix/COOL-1 knockdown (βPix-1 and βPix-2) on GBM U87R cell viability 3 days post kd. (**E**) The effect of beta-Pix knockdown (βPix-1 and βPix-2) on GBM cell survival using U87R-GFP cells in a colony formation assay (**F**,**G**) Flow cytometry analysis demonstrates an induction of apoptosis upon siRNA knockdown of βPix/COOL-1. A scrambled non-coding negative control (Cat#DSNC1, IDT Technologies, Coralville, IA, USA) was used as a control in all assays. Normalisation was performed by assigning the value for scramble control cells a value of 1 and expressing all other value relative to it. The data shown are arbitrary units (a.u.) means ± s.d. (*N* = 3). Two-way ANOVA, * *p* < 0.05, *** *p* < 0.001, two-tailed *t*-test.

We next assessed whether βPix/COOL-1 plays a role in other aspects of the malignant GBM phenotype. Specifically, to assess whether βPix/COOL-1 plays a role in U87R and GBM6 cell growth, we examined the effect of βPix/COOL-1 knockdown using the SRB colorimetric assay. We observed decelerated cell growth at 3 days post-knockdown, with a significant reduction (βPix-1: *p* = 0.0013; βPix-2: *p* = 0.0029) in U87R cell growth at 5 days post-knockdown in serum-containing conditions (Figure 3A). No significant reduction in growth was observed in GBM6 cells in serum-containing conditions (Figure 3B) at 3 days or 6 days post-knockdown (*p* = 0.171, *p* = 0.064 respectively). However, cells displayed a trend towards reduced proliferation 6 days post- knockdown. U87R cells were then assessed for proliferation in serum-free conditions and displayed a significant reduction (βPix-1: *p* = 0.001; βPix-2: *p* = 0.08) in proliferation at both 3 days and 5 days post-knockdown (Figure 3C). βPix/COOL-1 knockdown was also shown to significantly reduce the number of viable U87R cells (Figure 3D) present, with significant reductions in cell number at 3 days post-knockdown (βPix-1: *p* = 6.528 × 10^−5^; βPix-2: *p* = 0.0022). Depletion of βPix/COOL-1 did not significantly reduce (βPix-1: *p* = 0.2455; βPix-2: *p* = 0.099) the colony formation capacity observed in U87R cells, despite a trend towards reduced colony number (Figure 3E). Finally, the effect of βPix/COOL-1 knockdown on apoptosis was assessed in U87R cells using flow cytometry analysis of PI uptake (Figure 3F). In line with proliferation and viability assessments, βPix/COOL-1 knockdown significantly increased (*p* = 0.004) the number of PI-positive cells (i.e., cells that underwent primary or secondary necrosis and thus have ruptured cell membranes), indicating a significant increase in apoptosis at both early and later time points (72 h and 120 h respectively) (Figure 3G). Taken together, these data suggest that βPix/COOL-1 plays a role in pathophysiologic disease hallmarks including invasion, proliferation and survival.

### 2.4. βPix/COOL-1 Mediates Endothelial Cell Migration but Not Endothelial Tube Formation or Proliferation

**Figure 4:**
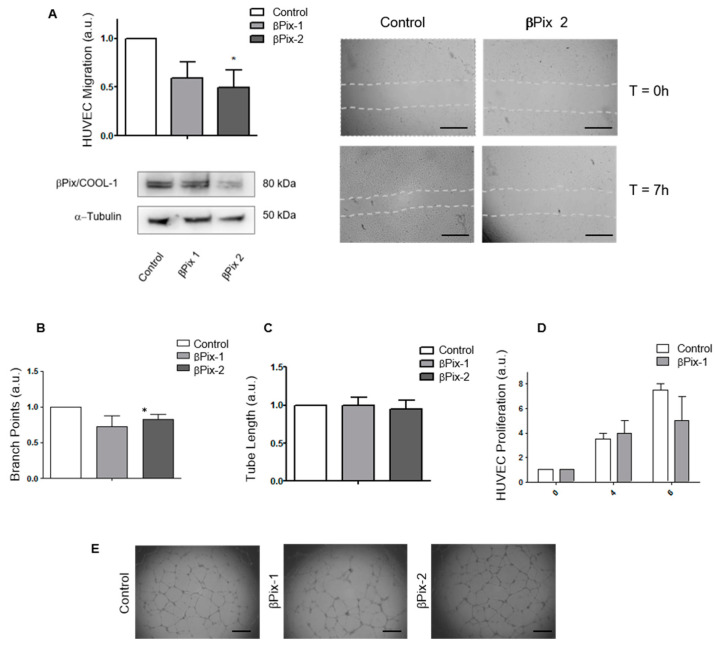
βPIX/COOL-1 mediates endothelial cell migration but does not play a role in endothelial cell tube formation or proliferation. (**A**) Wound closure was more complete in the control cells vs. the βPix/COOL-1 knockdown culture. 7 h post scratch there was a significant difference between scramble and βPix/COOL-1 knockdown cells. Western blot analysis confirms decrease in βPix/COOL-1 protein expression following siRNA knockdown (βPix-1 and βPix-2) in HUVEC cells. Tubulin was used as a loading control. (**B**–**D**) HUVEC branch points (**B**), Tube length (**C**) and Proliferation (**D**) were assessed with no significant differences observed. (**E**) Brightfield microscope images of tube formation assay in control and transfected cells. Scrambled non-coding negative control was used in all assays. All data were normalised to scramble control values. Data shown are arbitrary units (a.u.) with mean s ± s.d. (*N* = 3). Two-tailed *t*-test * *p* < 0.05. Scale bar (**A**) = 60 µm, (**E**) = 200 µm.

The effect of βPix/COOL-1 silencing on endothelial cell migration, tube formation and cell proliferation was next examined (Figure 4A–C). HUVEC cells were transfected with two βPix/COOL-1 siRNAs (βPix-1 and βPix-2, Figure S3A,B) as described, and the effect of βPix/COOL-1 knockdown on HUVEC cell migration using a wound healing assay was assessed. βPix/COOL-1 knockdown significantly decreased (βPix-1: *p* = 0.05; βPix-2: *p* = 0.0011) the migration of HUVEC cells with a 41.09% closure in cells transfected βPix/COOL-1-targeting siRNA, compared to 54.55% closure in control cells 7 h following wound generation (Figure 4A). To examine the effects of βPix/COOL-1 depletion on tubule function, we next employed an in vitro tubule-formation assay using HUVEC cells in which βPix/COOL-1 was silenced (Figure 4B–E). Interestingly, tubule branch point (βPix-1: *p* = 0.08999; βPix-2: *p* = 0.1765) and tubule length (βPix-1: *p* = 0.9453; βPix-2: *p* = 0.2825) were not significantly reduced following βPix/COOL-1 depletion (Figure 4B,C). Additionally, βPix/COOL-1 depletion did not significantly affect HUVEC cell proliferation at either 4 (*p* = 0.7591) or 6 (*p* = 0.8039) days following knockdown (Figure 4D). βPix/COOL-1 knockdown in HUVEC cells was confirmed via Western blot analysis (Figure S3). 

### 2.5. In Vivo Assessment of βPix/COOL-1 siRNA Loaded RGD-NP in Combination with Bev Indicates a Trend Towards Improved Survival

**Figure 5:**
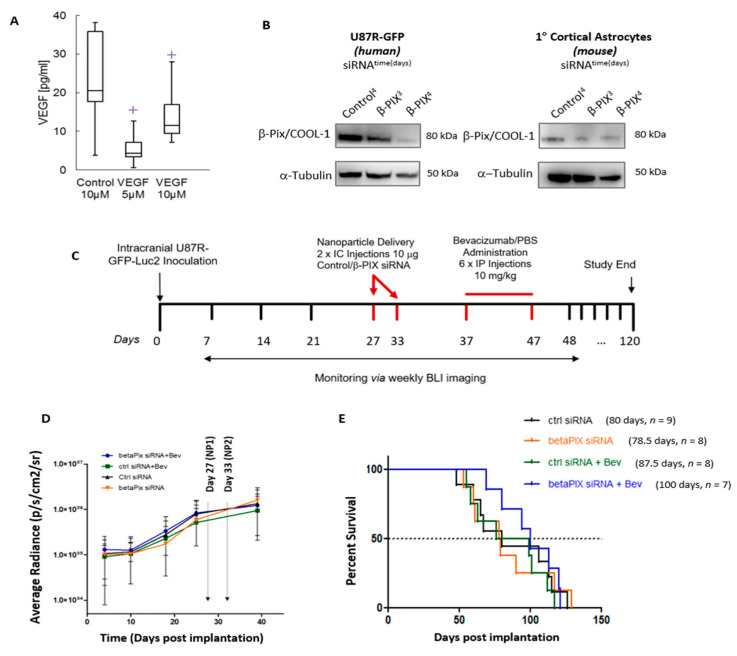
(**A**) Confirmation of in vivo efficacy of nanoparticle delivery system using VEGF targeting siRNA as a positive control. (**B**) Confirmation that βPIX/COOL-1 siRNA loaded RGD-NP delivered in vivo results in both human and mouse βPIX/COOL-1 knockdown. Western blot analysis confirms decrease in βPix/COOL-1protein expression following siRNA knockdown (βPix-1 and βPix-2) in human (U87R-GFP) and mouse (cortical astrocytes) cells. Tubulin was used as a loading control. (**C**) In vivo dosing regimen is presented. (**D**) Bioluminescence data showing U87R tumour growth across each experimental group. (**E**) In vivo data showing improved median survival for tumour bearing animals treated +Bev and βPIX/COOL-1 nanotherapeutic (*p* = 0.178 when compared) No significant difference was found when any groups were compared. Effect of treatment on survival using Kaplan–Meier analysis and log-rank (Mantel-Cox) tests was used to compare treatment groups. (Quantitative analysis of western blots could not be carried out as raw blots were unavailable in the archives).

In this proof-of-concept study, we employed an invasive, orthotopic GBM model to study the in vivo effect of the commercially available InVivoPlex targeted RGD-NP loaded with βPix/COOL-1 siRNA when delivered in combination with Bev. To first confirm that the RGD-NP was suitable for in vivo delivery of siRNA, VEGF-siRNA loaded RGD-NP was first delivered intracranially to *N* = 5 U87R-GFP-Luc2 tumour bearing mice. Efficient knockdown by VEGF-siRNA loaded RGD-NP was confirmed at 5 µM (*p* = 0.0083) and 10 µM (*p* = 0.0789) via ELISA assay (Figure 5A). Next, to confirm in vivo knockdown, βPix/COOL-1 siRNA-loaded RGD-NP was delivered intracranially to U87R-GFP-Luc2 tumour bearing mice. Subsequently βPix/COOL-1 protein levels in tumour cells and cortical astrocytes was assessed via western blot (Figure 5A). A decrease in βPix/COOL-1 protein expression was observed in U87R-GFP-Luc2 cells 4 days following in vivo βPix/COOL-1 transfection (Figure 5B). A minor decrease in βPix/COOL-1 expression was also observed in cortical astrocytes at day 3 and 4 following in vivo transfection. This confirms that delivery of βPix/COOL-1-siRNA using the RGD-NP results in efficient tumour target gene knockdown in vivo (Figure 5B) and minor off-target effects in cortical astrocytes (Figure 5B).

*N* = 40 NOD/SCID mice were intracranially implanted with U87R-GFP-Luc2 cells and tumour growth confirmed using bioluminescence (BLI) (Figure 5C). Following implantation one animal did not recover from surgery and seven animals did not form tumours (as confirmed by BLI). All remaining mice were randomised and treatment commenced on day 27 following implantation. βPix/COOL-1 targeting or scramble non-targeting siRNA (10 μg siRNA encapsulated in RGD-NP) was delivered locally to these tumours via intracranial injection into the tumour site, with mice receiving two injections at one-week intervals. Three days following the second administration of RGD-NP, animals were treated with Bev or phosphate buffered saline (PBS) vehicle control via intraperitoneal injection (6 × 10 mg/kg every second day over 12 days). The study design is illustrated in Figure 5C. Tumour growth was monitored for 5 weeks post implantation using imaging (BLI) (Figure 5D). No significant differences in BLI were observed between groups at any timepoint (*p* = 0.9571). Survival analysis showed that animals receiving Bev (+scramble siRNA-loaded RGD-NP control) had a median survival of 88 days, when compared with control animals (scramble siRNA-loaded RGD-NP + PBS Vehicle control) with a median survival of 80 days (*p* = 0.571). Mice treated with βPix/COOL-1-siRNA loaded RGD-NP (+PBS Vehicle control) had a median survival of 78.5 days (vs. PBS Vehicle control *p* = 0.845), indicating that βPix/COOL-1 targeting alone does not confer any survival benefit (Figure 5E). Finally, animals treated with both Bev and βPix/COOL-1 siRNA-loaded RGD-NP showed a trend towards increased survival (*p* = 0.1783), having an improved median survival of 100 days when compared with other treatment groups.

## 3. Discussion

GBM morbidity and mortality is largely due to rapid tumour growth, neovascularization, and tumour invasion into the brain parenchyma [38,39]. Options for targeting these disease hallmarks are limited, and no adjuvant anti-invasion therapeutic strategies currently exist. Nevertheless, targeting GBM neovascularization is acknowledged as a rational therapeutic intervention. In this respect, Bev has been approved by the US Food and Drug Administration (FDA) for the treatment of GBM. Conversely this approval has not been recommended by the European Medicines Agency (EMA) due to lack of overall survival benefits [7] and evidence that in virtually all cases, Bev treated GBM patients develop resistance with some tumours developing increasingly aggressive phenotypes [40]. Indeed, anti-angiogenic therapies have been shown to enhance the invasion of GBM tumours [14] and distant infiltrative disease may be observed following treatment [16]. As previously suggested, adjuvant therapies designed to target multiple cancer hallmarks represents a rational approach [31,41]. Therefore, we hypothesised that targeting Rho GTPase signaling could act as a combined anti-invasive/anti-angiogenic therapeutic strategy, as RhoGEFs are mediators of key components in pathways which underpin tumour and Bev-induced invasion [22], and RhoGEF is involved in anti-angiogenic resistance pathways.

We screened the GEF protein family (comprised of 83 members) for altered gene expression in a publicly available panel (GSE12689) of treatment naïve GBMs. In this dataset we identified that the GEF βPix/COOL-1 was significantly upregulated in the leading tumour edge when compared to the tumour core. βPix/COOL-1 mRNA expression was further assessed using the IvyGAP database (GSE107559), comprising of *N* = 42 tumours from *N* = 41 patients. Using laser capture microdissection, samples within this database were delineated to define the tumour leading edge, infiltrating tumour, cellular tumour, pseudopalisading cells around necrosis, and microvascular proliferation [34] allowing the assessment of gene expression in these defined tumour locations. βPix/COOL-1 mRNA was significantly upregulated in the leading edge and infiltrating tumour samples compared to the cellular tumour region, further highlighting the potential role of βPix/COOL-1 in driving tumour infiltration. Unfortunately, as annotated tissue either from the Hoelzinger et al. [33] and Puchalski et al. [34] studies or elsewhere was not available, we were unable to orthogonally validate these findings at the protein level. It is noteworthy that tumour sample annotation of core vs. leading/infiltrative edge is not widely available in standard tumour biopsy samples (e.g., TCGA). Indeed, large numbers of such annotated datasets are outstanding in the field and would certainly be useful in the development of targeted anti-invasive therapies. Nevertheless, the endogenous protein expression of βPix/COOl-1 in the U87R invasive GBM cell line is shown in western blots contained in Figure 2 and Figure 5, and in cortical astrocytes in Figure 5.

βPix/COOL-1, (encoded by the ARHGEF7 gene), is a member of the PIX family proteins which have important functions in several disease settings [42,43]. βPix/COOL-1 acts as a GEF protein for the Rho family of small GTP-binding protein family members Rac1 and Cdc42. βPix/COOL-1 forms several protein complexes resulting in a number of downstream phenotypic changes including cell migration (P-cadherin complex; via CDC42), leading edge polarization (SCRIB complex; via CDC42 and PAK), focal adhesion turnover (PAK and GIT complex; via paxillin and Rac1) and formation of invadopodia (Gαi2 complex; via SRC and RAC1 signaling) [23]. Additionally, βPix/COOL-1 has been shown to bind to c-CBL, an E3 ubiquitin ligase, playing a key role in the downregulation of the EGFR receptor. This binding of and sequestration of c-CBL by βPix/COOL-1 prevents c-CBL induced EGFR degradation, resulting in sustained EGFR expression [44]. Broadly, the gene has also been shown to play a role in the formation of focal adhesions, mediating cancer cell response to ECM interaction [45], synaptic structure development, neuronal polarization in the early stages of axon formation [46] and cell migration [47]. The role of βPix/COOL-1 in the cancer setting has also been studied [48,49]. For example, a recent study assessed the expression of βPix/COOL-1 in patient derived primary and secondary GBM cell lines. Here, several βPix/COOL-1 isoforms were shown to be upregulated in neoplastic brain tissues, when compared to normal cortex expression levels. Moreover shRNA-mediated depletion of βPix/COOL-1 sensitized GBM cell lines to carmustine (BCNU) and TMZ and inhibited multicellular spheroid formation in vitro [48].

Overall, our data (along with previously published work) provided a rationale to further study βPix/COOL-1 as a potential anti-invasive therapeutic target in GBM. In this context, we first demonstrated that siRNA-mediated depletion of βPix/COOL-1 in the invasive U87R and GBM6 GBM cell lines result in decreased cell invasion in 3D models, reduced cell proliferation, diminished colony forming capacity and induction of apoptosis. These data suggest a role for βPix/COOL-1 in supporting the invasive phenotype common to GBM. Interestingly, these data align closely with data for other GEFs such as GIT1 which when silenced via siRNA (or indeed miR-149) was shown to decrease invasion and metastases in the setting of triple negative breast cancer [50]. βPix/COOL-1 has also been shown to interact with PAK1 (p21-activated kinase) and STIL (SCL/TAL1-interrupting locus), to promote actin remodelling and promote cancer cell migration in pancreatic cancer models [51] suggesting a potential mechanism for the βPix/COOL-1 mediated invasion evident in GBM. Employing a wider panel of invasive GBM cell lines in future studies would further support these findings. Additionally, studies which assess the impact of βPix/COOL-1 overexpression on GBM invasion and proliferation (such those performed by Lei et al. [49] in the colorectal cancer setting) would be of additional interest.

To assess the role of βPix/COOL-1 gene silencing on angiogenesis, we next studied the impact of βPix/COOL-1 siRNA silencing on HUVEC cell function. Notably, analysis of GSE84465 for ARHGEF7mRNA expression displays a trend towards increased expression in vascular cells compared to other cell types, thus supporting the hypothesis that βPix/COOL-1 may play a role in GBM endothelial cell function. We observed that knockdown of βPix/COOL-1 inhibits HUVEC cell migration, but not HUVEC tube formation or proliferation. These results suggest that βPix/COOL-1 function in angiogenesis is not related to increased endothelial cell proliferation, but rather may facilitate the migration of endothelial cells. Overall these data reflect previous findings where GEFs such as Syx, TEM4 and Dia1 are shown to have a critical roles supporting endothelial cell organization and endothelial junction integrity [52]. Our findings also align with a previous report which suggest that the GEFs βPIX/COOL-1 and Tiam1 mediate Rac-dependent endothelial barrier regulation [53]. Studies to further interrogate the role of βPix/COOl-1 in the endothelium, could incorporate Annexin V-FITC/PI staining following βPix/COOl-1 knockdown to assess the effect of βPix/COOl-1 depletion on endothelial cell apoptosis. Additionally, endothelial survival could be assessed in future studies via sulphorhodamine B assay following βPix/COOl-1 depletion.

Finally, in a proof of concept study we employed an orthotopic disease model to assess the effect of a novel combinatorial treatment regimen comprised of Bev and a nanoparticle gene silencing system (RGD-NP) loaded with βPix/COOL-1 targeting siRNA. In vivo data demonstrated that βPix/COOL-1 targeting alone does not confer a significant survival benefit when compared with Bev or vehicle. Nevertheless, our data suggests a trend towards improved median survival in mice receiving combination therapy with both Bev and βPix/COOL-1-siRNA loaded RGD-NP (median survival increase of 12 days in animals receiving combination therapy compared to control or Bev alone *p* = 0.1783). Interestingly, knockdown of βPix/COOL-1 in GBM cells was previously shown to prevent tumour formation in GBM21- and GBM27-NOD/SCID orthotopic models [48]. βPix/COOL-1 has also been shown to promote the development of metastases in colorectal adenocarcinomas, with high levels of expression associated with a more aggressive phenotype [49].

To build on this proof of concept work, additional adequately powered preclinical studies of βPix/COOL-1 depletion are now warranted. These studies should implement faithful, orthotopic patient derived xenograft (PDX) models and incorporate standard of care (TMZ, radiation, resection) treatment. Studies to compare the effects of silencing βPix/COOL-1 both in the neoadjuvant and adjuvant setting are also warranted. It is also likely that a more ‘refined’ NP targeting strategy could improve outcome; either by improving selectivity to tumour cells or by enhancing siRNA delivery. In this study, we implemented a generic NP targeted siRNA βPix/COOL-1 gene silencing approach. The RGD conjugated InVivoPlex (AparnaBio) NP system (RGD-NP) used was a non-imageable, non-customized entity. Future studies could likely benefit from the delivery of βPix/COOL-1 siRNA in an optimized and customized NP system which is targeted to tumour cells alone. For example, we have recently designed [54] a novel, targeted polymeric siRNA nanocarrier system, modified which is specifically customized for the delivery of gene specific siRNA in vivo. Additionally, recent studies point to ionizable lipids as superior excipients for siRNA delivery [55]. Overall, imageable, targeted theranostic nanotherapeutics are superior to commercially available systems and support more efficient and direct siRNA delivery to brain tumours [56,57].

## 4. Materials and Methods

### 4.1. Identification of RhoGEFs Differentially Expressed in the Leading Edge of Patient Tumours

Publicly available gene expression array data (NCBI Gene Expression Omnibus GSE12689) [33,58], was analysed to identify RhoGEFs with increased expression in the invasive tumour edge compared with the tumour core for a panel of 19, treatment naïve, GBM tumours. Expression values were filtered and RhoGEF expression at the tumour rim was normalized to RhoGEF expression at the tumour core. RNA-Seq dataset GSE107559 (Ivy Glioblastoma Atlas Project [34]) was analysed to validate the findings within GSE12689. The GlioVis data portal (http://gliovis.bioinfo.cnio.es) [59] was used for the analysis of this validation dataset.

### 4.2. Cell Culture

The invasive human GBM cell line U87R-GFP (a gift from Peter Forsyth, Moffitt Cancer Center, Tampa, FL, USA) was cultured in Dulbecco’s Modified Eagle’s Medium (DMEM) F12 supplemented with 10% fetal bovine serum and 400 mg/mL G418 as previously described [36]. Human umbilical vein endothelial cells (HUVECs) were cultured in complete Endothelial Cell Basal Medium 2 (EBM-2). The GBM patient-derived xenograft (PDX) cell line, GBM6 (a gift from Jann Sarkaria, Mayo Clinic, Rochester, MN, USA) was established from GBM tumour material serially passaged in mice, as previously reported [37]. The GBM6 line was cultured on poly-L-lysine and laminin coated plastic ware, in Knockout DMEM-F12 media (Gibco, Waltham, MA, USA) supplemented with EGF, FGF and StemPro (Gibco). Cells were maintained at 37 °C and 5% CO_2_. We acknowledge the growing problem of cell line misidentification which has been highlighted in several cases in the literature. We note that recent literature indicates the U87MG cell line no longer reflects the original tumour from which it was derived and harbours a different genotype to that of the originally isolated patient cells. Nevertheless, the literature does indicate that the U87 cell line remains classified as a GBM tumour-derived cell line, despite the genomic differences now present [60].

### 4.3. siRNA Transfections

Cells were transfected using 2 separate siRNA duplex molecules that target βPix/COOL-1 mRNA, and as a control, a scramble siRNA duplex that targets no known mRNA. siRNA duplexes for βPix/COOL-1were purchased from IDT (Coralville, IA, USA). 3 × 10^5^ cells were seeded in each well of a 6-well cell-culture plate in standard growth medium for all assays. After 5 h, dharmafect 1 (2 μL, Dharmacon, Inc., Lafayette, CO, USA) in 200 μL Opti-MEM (Gibco–Life Technologies Corp., Norwalk, CT, USA) was mixed with siRNA duplex (5 nM concentration) in 200 μL Opti-MEM and incubated for 20 min. Media were then removed from cells and 400 μL of siRNA/Opti-MEM mixture was added dropwise to cellsand 1.6 mL antibiotic-free medium was added. 24 h later medium was removed and replaced with fresh antibiotic-containing. Viability of cells were assessed via trypan blue assay 2 days following transfection and cells passaged for further use [61]. The following siRNAs (sense strand provided) were used at 5 nM concentration: βPix/COOL-1 duplex #1: 5′-AGCCUUCAGAUGAGGAGUUCGCGTC-3′ (βPix-1), and βPix/COOL-1 duplex #2: 5′-AUCAUACA GAUAGACAAGAUAUUCA-3′ (βPix-2). A non-targeting negative control was also used (Catalogue #DS NC1).

### 4.4. Western Blot Analysis of βPix/COOL-1 Expression

βPix/COOL-1 protein levels in whole cell lysates were determined using western blot analysis. Following treatment, cells were washed (PBS)cells lysed with RIPA buffer (Cell Signalling Technology, Inc., Danvers, MA, USA) master mix (containing PhosSTOP protease and phosphatase inhibitor cocktails). Total protein concentration was determined via Bradford assay. SDS–PAGE was then used to separate proteins (equal amounts per well) and proteins transferred onto PVDF membrane. Specific proteins were then detected using βPix/COOL-1 specific antibody (07-1450, Millipore Inc., Darmstadt, Germany) and α-tubulin antibody (Clone DM1A, T6199 Sigma-Aldrich, Sigma Aldrich, Gillingham, UK). Membranes were developed by ECL and films scanned (Figure 2, Figure 4, Figure 5, Figures S2 and S3). Densitometric analysis was performed using ImageJ software (National Institutes of Health, Bethesda, MD, USA).

### 4.5. Sulpho-Rhodamine B (SRB) Proliferation Assay

In all, 5000 cells were added per well of a 96 well plate (in duplicate) in FBS-free medium. Cells were cultured under standard conditions following transfection, changing the medium every 2 days. Cells were fixed at 4 h, 4 days and 6 days post seeding using 100 μL 10% trichloroacetic acid (TCA) in PBS at 4 °C for 60 min. Each well was washed with deionized water and allowed to dry. 100 mL of 0.2% sulforhodamine B (SRB) in 1% acetic acid was added to each well and agitated at 300 r.p.m. for 30 min at room temperature. Each well was washed in 1% acetic acid and allowed to dry. 200 µL of 10 mM Tris was added to each well and agitated at 300 r.p.m. at room temperature for 30 min before absorbance was read at 490 nm using a plate reader.

### 4.6. Cell Cycle Analysis of GBM Cells

72 h and 120 h post-transfection medium was collected and adherent U87R cells in monolayer culture harvested, washed twice with sample buffer (100 mg glucose; 100 mL PBS without Ca^2+^ or Mg^2+^) and fixed in 70% (*v*/*v*) cold ethanol. Cells were pelleted, washed once with sample buffer and resuspended in propidium iodide (PI) solution (50 μg mL^−1^ PI, 0.5 mg mL^−1^ RNase in sample buffer, pH 7.4) for 30 min in the dark. The data from 10,000 cells per sample were collected and analysed using the BD FACSDiva program on a LSR II HTS flow cytometer (BD Bioscience, Franklin Lakes, NJ, USA).

### 4.7. Matrigel Invasion Assay

Freshly transfected cells were incubated for 16 h in serum-free medium. Cells were detached using accutase (#A6964, Sigma-Aldrich) and counted using a haemocytometer. 1 × 10^5^ cells were resuspended in 50 μL of basement membrane extract (BME, thawed at 4 °C) diluted to 10 μg mL^−1^ with serum-free media. This mixture was added to the 24-well transwell insert. The BME-cell mixture, was allowed to polymerise for 30 min by incubation at 37 °C. In all, 200 μL of serum free medium was added to the upper chamber and 700 μL of media supplemented with 20 serum, was added to the lower well. Cells were allowed to invade for 24 h at 37 °C and 5% CO_2_. Chambers were placed in 4% (*v*/*v*) paraformaldehyde (PFA) in PBS for 30 min at room temperature followed by crystal violet solution (0.25% *w*/*v*) for 40 min at room temperature. Chambers were washed in PBS and a cotton swab was used to remove non-invading cells were from the insert. Inserts were allowed to dry and were imaged using a Zeiss Axiovert 200M microscope (Zeiss, Oberkochen, Germany) equipped with a 2.5× objective. Invading cells were counted manually and using the Image J software.

### 4.8. Migration Assay

To observe the wound-healing response of HUVEC cells, transfected cells were seeded in duplicate in a 6-well plate. Subsequently once cells became confluent (day 3), a wound healing migration assay was performed by making three parallel scratches in the each of the six wells using a sterile 10 µL micropipette tip. Cells were washed and 500 μL fresh serum-free medium added. Images of each scratch were taken using a light microscope at time 0 h, 3 h and 7 h post scratch. Using Image J software, distances were measured at 6 different points on each and were plotted as percentage migration from time 0 h.

### 4.9. Clonogenic Assay

To assess clonogenic survival of cells in monolayer culture, cells were transfected with βPix/COOL-1 siRNA or scramble siRNA as described above. Cells were then plated in triplicate at 500 cells per well, into six-well cell culture plates in serum containing media. Colonies were allowed to grow for 10 days. Colonies were fixed with 4% PFA and stained with crystal violet (0.25% *w*/*v*). Colonies containing >50 cells were counted via light microscopy to determine percent survival Three replicates were averaged for each treatment.

### 4.10. Tube Formation (Angiogenesis) Assay

In order to assess the effect of βPix/COOL-1 knockdown on tube formation, following transfection, 1.5 × 10^4^ HUVECS were added to matrigel coated wells of a 96 well plate in 150 μL of complete medium. Cells were then incubated for 20 h at 37 °C. Images were taken using light microscope and analysed with ImageJ. The number of branching points (the point at which a tube splits) were counted as were the lengths of each tube from branching point to branching point.

### 4.11. Enzyme-Linked Immunosorbent Assay

In order to confirm the efficacy of the commercially available (Arg-Gly-Asp (RGD)-conjugated, InVivoPlex, AparnaBio, Rockville, MD, USA) nanoparticle system (RGD-NP), we aimed to first assess VEGF knockdown in U87 tumour material. Tissue was harvested from mice treated with VEGF siRNA-loaded RGD-NP (5 µM or 10 µM) and homogenized in RIPA lysis buffer containing protease inhibitors. Tissue homogenate was centrifuged at 13,000 rpm for 10 min at 4 °C to pellet insoluble contents and collect the supernatant. The supernatant was then assessed using the VEGF ELISA Kit (R&D Systems, Abingdon, UK) which detects human VEGF-A, including VEGF165, human VEGF121, and human VEGF165b.

### 4.12. GBM Orthoxenograft Studies

In vivo studies were licensed and approved by the Department of Health and Children Dublin, Ireland (License number B100/3654). Protocols were further approved by the Royal College of Surgeons in Ireland Animal Research Ethics Committee. Animal experiments were carried out under Directive 2010/63/EU of the European Parliament on the protection of animals used for scientific purposes. All in vivo studies were carried out in a specific pathogen free (SPF) facility. Adult NOD/SCID mice (Female) were imported from Charles River UK (Cambridge, UK) housed in groups of *N* = 4 in individually ventilated cages (Techniplast, London, UK) and given access to food and water ad libitum. Environmental enrichment of red polycarbonate houses, nesting material and clear plastic tunnels was provided. A 12 h light/12 h dark cycle, 40–50% humidity and temperature of 18–22 °C were maintained throughout. Animals were assessed daily to monitor overall health status. Experimental animal numbers were calculated using the formula: *N* = (*Z*α + *Z*β)^2^ × (2σ^2^/δ^2^).

Throughout, female NOD/SCID (4–6 weeks; 18–22 g average weight) mice were anesthetized with O_2_/isoflurane mixture (1.5% isofluorane in 100% O_2_), and 2 × 10^5^ of U87R-GFP-Luc2 cells orthotopically implanted via stereotaxic injection as previously described [62]. Bioluminescence imaging (BLI) performed with an IVIS Spectrum (Perkin Elmer, Waltham, MA, USA) was employed to monitor tumour growth until treatment commenced. Imaging was carried out under anesthesia (1.5% isofluorane in 100% O_2_) on a heated stage. 15 min prior to imaging mice received150 mg/kg luciferin (Perkin Elmer) administered via intraperitoneal injection. A 1 s reference image was taken (binning = 4, F-stop = 1). Living Image software (V4.3.1, Perkin Elmer Perkin Elmer, Waltham, MA, USA) was utilised for image analysis and average radiance (p/s/cm^2^/sr) was analysed.

In all studies, prior to commencing therapy mice were randomized into treatment groups using the = Rand() function in Excel, and treatment group identifiers blinded. All intratumoural injections were carried out under anesthesia delivered via nose cone (1.5% isofluorane in 100% O_2_), in a stereotaxic frame using non-rupture ear bars (World Precision Instruments, Sarasota County, FL, USA). Animals were maintained on a heated bed and temperature monitored throughout. Adverse effects were scored on a multi-category scale and a modified rodent coma scale employed to monitor neurological status. Animals were monitored daily for adverse effects of treatment.

Confirmation of in vivo knockdown capacity of the siRNA-loaded RGD-NP was carried out prior to commencement of the efficacy study. Tumour-bearing mice (*N* = 5) received an intratumoural injection of RGD-NP loaded with βPix/COOL-1 specific siRNA (10 µg) using a 5 μL Hamilton syringe, and were euthanised via cervical dislocation on day 3 and 4 following injection. U87R tumour cells and cortical astrocytes were macro-dissected from the bulk of the brain tissue, and protein extracted. western blot analysis was performed as described above (Figure S4).

For the efficacy study, *N* = 40 mice were randomized into four treatment groups 2 weeks following implantation. βPix/COOL-1 targeting siRNA or scramble control siRNA were delivered locally to tumours using the RGD-NP via a 5 μL Hamilton syringe. Mice received two doses of siRNA-loaded RGD-NP (10 µg siRNA) on day 27 and 33 following tumour implantation. On day 37 following implantation animals were treated with Bev or Vehicle (PBS) (6 × 10 mg/kg IP, once every second day). For survival analysis animals were humanely euthanised via cervical dislocation upon presentation of neurological symptoms (ataxia, staggering paralysis, seizure or head tilt) or >20% weight loss. All sections of this manuscript adhere to the ARRIVE Guidelines for reporting animal research. 

### 4.13. Statistical Analysis

All analyses were performed in triplicate. Data are expressed as means with error bars representing standard deviation (SD). Data were analysed using GraphPad software (GraphPad Software, San Diego, CA USA) or Excel (Microsoft, Redmond, WA, USA). A Student’s *t*-test, Wilcoxon signed rank test and ANOVA was performed to assess statistical significance with *p* < 0.05 deemed significant. Statistical analysis of the in vivo data was performed using a log-rank test for survival studies with *p* < 0.05 deemed significant.

## 5. Conclusions

Our work presents proof-of-concept evidence that the RhoGEF βPix/COOL-1 might represent a vulnerable therapeutic target in GBM. A limitation of the current study was a lack of funding to study the effect of a targeted NP system to deliver βPix/COOL-1 siRNA in combination with Bev in additional clinically relevant animal models. Future studies should employ large cohorts of faithful preclinical models (e.g., PDX population trials [63]) and incorporate standard of care therapy (TMZ, radiation). It will also be important to assess therapeutic efficacy in the adjuvant or recurrent setting, using models which support tumour resection [63,64].

## Supplementary Materials

The following are available online at https://www.mdpi.com/2072-6694/12/12/3531/s1, Figure S1: Rim/core ration of top genes from GSE12689 dataset which were predominantly overexpressed in the tumor rim, Figure S2: Western blot and densitometry analysis (of three independent replicates) showing βPix/COOL-1 proteinn expression following siRNA knockdown in (A) U87R-GFR cells and (B) GBM6 confirms knockdown. Extended blots demonstrating specificity of antibody, Figure S3: Western blot analysis showing βPIX/COOL-1 protein expression following siRNA knockdown HUVEC cell. Figure S4: Western blot and densitometry analysis showing βPix/COOL-1 protein expression following siRNA knockdown in cortical astrocytes at 3 and 4 days post-knockdown.
