# Supplementary material for: Targeting the RhoGEF βPIX/COOL-1 in Glioblastoma: Proof of Concept Studies
Source: Cancers (Basel). 2020 Nov 26;12(12):3531. doi: 10.3390/cancers12123531 (PMC7761123; doi:10.3390/cancers12123531)
Supplement: Supplementary file 1 [file cancers-12-03531-s001.pdf]

Supplementary Materials: Targeting the RhoGEF βPIX/COOL-1 in Glioblastoma: Proof of Concept Studies

Kate Connor, David W. Murray, Monika A. Jarzabek, Nhan L. Tran, Kieron White, Patrick Dicker, Kieron J. Sweeney, Philip J. O'Halloran, Brian MacCarthy, Liam P. Shiels, Francesca Lodi, Diether Lambrechts, Jann N. Sarkaria, Raymond M. Schiffelers, Marc Symons and Annette T. Byrne

A

	44 JAP	49 JAP	BNI-364	BNI-370	BNI-660	BNI-758	BNI-839	BNI-9065	BNI-9076	BTTB1020	BTTB886	BTTB965	TB186	TB187	TB195	TB197	VPJT-01	VPJT-03	VPJT-07 D
KALRN	60.77	0.23	11.36	0.59	6.28	3.44	2.58	2.26	8.90	16.79	79.08	1.93	10.05	3.33	31.02	7.91	2.66	0.70	14.03
MCF2	10.33	1.59	3.14	1.26	4.48	15.52	1.37	6.05	4.96	2.45	4.43	1.25	2.06	0.70	4.46	3.54	5.49	2.65	1.53
NGEF	2.23	0.47	10.19	1.66	6.94	4.11	1.36	1.72	9.92	5.79	6.03	1.01	2.74	0.57	25.92	6.72	9.66	2.54	17.26
ARHGEF7	14.92	1.14	0.51	0.58	1.75	1.73	2.05	3.32	4.12	2.31	5.48	3.17	3.76	0.95	7.26	1.63	3.02	0.55	0.63
CDC42EP2	3.83	1.69	1.21	1.54	2.36	2.35	1.79	1.49	0.44	4.27	2.61	1.17	0.79	3.35	2.06	2.36	1.94	1.54	0.34
DOCK9	8.96	2.58	1.87	2.09	0.95	1.13	2.22	0.78	1.78	2.87	3.29	1.00	2.11	0.78	8.35	2.26	1.92	1.32	1.52
NET1	1.97	1.67	0.69	1.03	1.76	8.50	1.52	10.47	1.46	7.62	2.83	0.99	3.88	3.09	2.99	1.39	5.09	0.90	10.22
TIAM2	49.03	0.97	6.00	1.25	2.88	2.08	0.89	23.39	0.80	4.82	3.20	1.14	2.32	3.26	3.24	5.24	1.64	0.84	9.59
ABR	5.01	1.33	4.18	0.92	2.73	1.79	0.66	0.90	2.18	6.09	2.13	1.06	6.24	1.03	2.79	1.85	2.48	0.81	3.09
PLEKHG5	1.65	0.73	0.89	0.73	0.94	1.12	2.44	0.93	1.39	5.89	6.51	1.25	2.36	1.83	6.11	5.95	1.76	1.13	2.02
RHOBTB2	0.72	1.42	2.04	0.89	3.51	2.16	0.93	3.37	0.83	4.69	3.31	1.16	1.70	0.80	2.61	1.28	1.76	2.19	1.14
PREX1	5.79	1.58	1.86	1.49	4.45	1.05	1.82	0.35	0.73	0.95	0.51	1.00	1.60	2.03	1.47	1.65	1.09	1.53	0.38

>1.5	<1.5	<0.70

Figure S1. Rim/core ration of top genes from GSE12689 dataset which were predominantly overexpressed in the tumor rim.

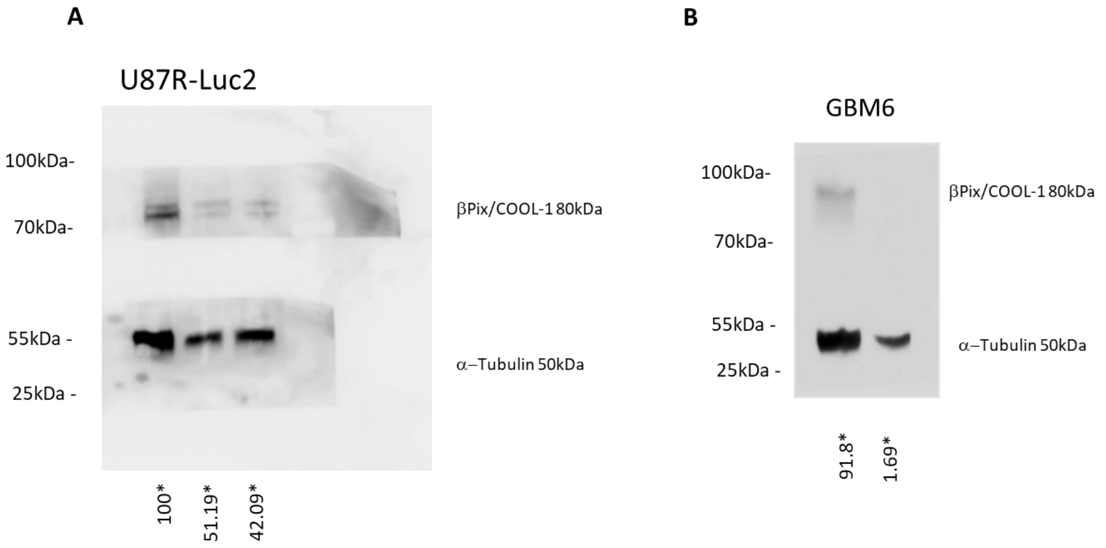

Figure S2. Western blot and densitometry analysis (of three independent replicates) showing βPix/COOL-1 proteinn expression following siRNA knockdown in (**A**) U87R-GFR cells and (**B**) GBM6 confirms knockdown. Extended blots demonstrating specificity of antibody.

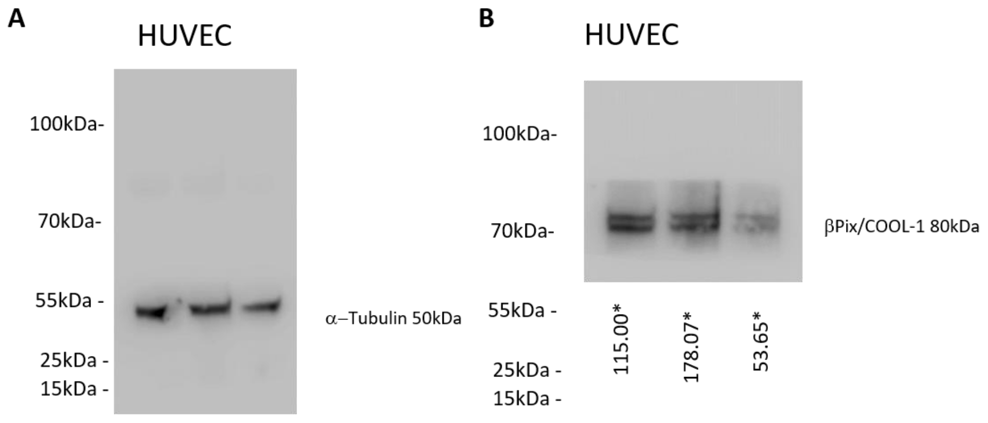

*Intensity after normalisation to loading control

Figure S3. Western blot analysis showing βPIX/COOL-1 protein expression following siRNA knockdown HUVEC cell. (**A**,**B**) Extended a Tubulin and βPIX/COOL-1 bolt demonstrating specificity of antibody. All βPix/COOL-1 band intensities are normalised to relative a Tubulin band. Prior to imaging of the βPIX/COOL-1 antibody signal, western blots were cut to remove the anti-a tubulin bands.

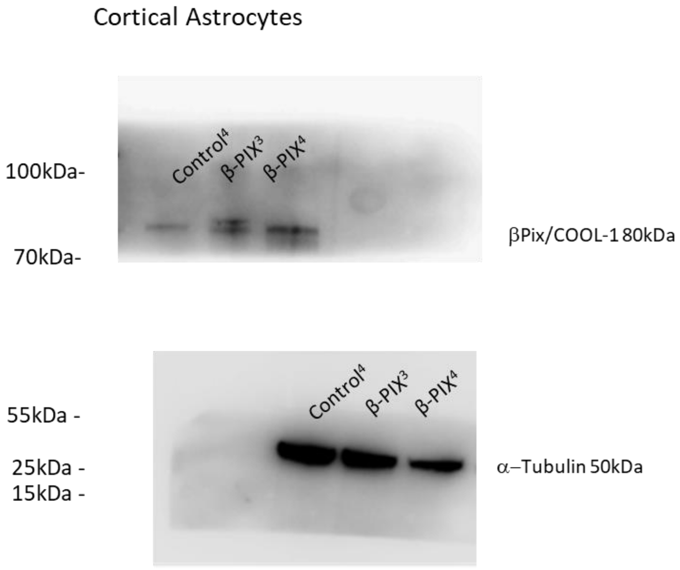

Figure S4. Western blot and densitometry analysis showing βPix/COOL-1 protein expression following siRNA knockdown in cortical astrocytes at 3 and 4 days post-knockdown. Prior to probing with each antibody (βPix/COOL-1 /a-Tubulin), the membranes were cut.

Publisher's Note: MDPI stays neutral with regard to jurisdictional claims in published maps and institutional affiliations.

© 2020 by the authors. Licensee MDPI, Basel, Switzerland. This article is an open access article distributed under the terms and conditions of the Creative Commons Attribution (CC BY) license (http://creativecommons.org/licenses/by/4.0/).
